# Effects of an enzyme complex on in vitro dry matter digestibility of feed ingredients for pigs

**DOI:** 10.1186/s40064-015-1060-1

**Published:** 2015-06-17

**Authors:** Changsu Kong, Chan Sol Park, Beob Gyun Kim

**Affiliations:** Department of Animal Science and Technology, Konkuk University, Seoul, 143-701 Republic of Korea

**Keywords:** Enzymes, In vitro dry matter digestibility, Non-starch polysaccharide, Pigs

## Abstract

Feed ingredients of plant origin are commonly used in swine diets. However, the major components of plant cell walls, non-starch polysaccharides (NSPs), reduce nutrient digestibility. To improve the efficiency of feed utilization, exogenous enzyme products that degrade NSPs have been widely used in commercial animal feeds. Nonetheless, the effects of exogenous enzyme addition to swine diets on nutrient digestibility have not been determined. To this end, in vitro approaches may be used. The objective of this study was to determine the effects of an enzyme complex (EC) containing xylanase, protease, and phytase on the in vitro dry matter (DM) digestibility of nine feed ingredients including cereal grain energy sources (corn, wheat, and barley) and protein sources (soybean meal, rapeseed meal, palm kernel meal, cottonseed meal, copra meal, and distillers dried grains with solubles). Both in vitro ileal and total tract digestibility (IVID and IVTTD, respectively) of DM were determined for the nine test ingredients, with or without EC addition. The EC addition increased the IVID of DM in copra meal (*p* = 0.047) and tended to increase the IVID of DM in corn, wheat, barley, palm kernel meal, cottonseed meal, and DDGS (*p* < 0.10). On the other hand, no significant effect was observed in soybean meal and rapeseed meal. The IVTTD of DM in the test ingredients was not affected by the addition of EC, except for cottonseed meal (52.1 vs. 50.6%, *p* = 0.053). In conclusion, the effects of EC addition on in vitro DM digestibility may vary, depending on the test ingredient and method used.

## Background

Feedstuffs of plant origin are widely used in swine diets. However, the major components of plant cell walls, non-starch polysaccharides (NSPs), have anti-nutritive effects on nutrient digestibility. Arabinoxylan, a cell wall NSP consisting of arabinose and xylose (Saulnier et al. 2012), is the most common pentosan in cereal grains such as corn, wheat, and barley (Masey O’Neill et al. 2014). The use of exogenous enzymes in swine diets is a common strategy to ameliorate anti-nutritive effects of NSPs on nutrient utilization. Amongst these, xylanase and glucanase are dominant NSP-hydrolyzing enzymes in swine feeds and their effects have been extensively studied (Adeola and Cowieson 2011). Furthermore, because phytates are generally concentrated in fibrous cell wall in a practical swine diet, a cocktail of multi-carbohydrase and phytase was also proposed to improve nutrient digestibility of pigs (Kiarie et al. 2010).

In vitro digestibility methods that simulate the gastrointestinal condition of pigs have been used to predict the apparent ileal and total tract digestibility of nutrients in various feedstuffs (Boisen and Fernández 1995, 1997; Regmi et al. 2009; Park et al. 2012; Cervantes-Pahm et al. 2013), and to test the efficacy of mycotoxin sequestering agents (Kong et al. 2014). It is therefore possible that this approach can be similarly applied to determine the efficacy of exogenous enzyme addition to swine diets on nutrient digestibility. However, these methods have not been evaluated for estimating digestibility of nutrients in swine feed or ingredients containing exogenous enzymes. Therefore, the objective of the present study was to determine the efficacy of an enzyme complex (EC) containing xylanase, protease, and phytase, on in vitro ileal and total tract digestibility (IVID and IVTTD, respectively) of dry matter (DM) in ingredients for pigs.

## Methods

### Enzyme complex and test ingredients

The EC contained a combination of *Aspergillus niger*-derived xylanase, protease and phytase at 100, 700 and 300 units/g of complex, respectively. Nine feed ingredients were used for in vitro DM digestibility. These included corn, wheat, and barley as energy sources, and soybean meal, rapeseed meal, palm kernel meal, cottonseed meal, copra meal, and distillers dried grains with solubles (DDGS) as protein sources.

### Sample preparation

Before the analyses, ingredients were finely ground and each ingredient was divided into two groups. Each group was supplemented with either EC or wheat bran at 1.0%. After mixing, three ingredient samples from each group were weighed for in vitro DM digestibility analyses.

### In vitro digestibility procedures

In vitro procedures were modified from a two-step IVID (Boisen and Fernández 1995) or three-step IVTTD (Boisen and Fernández 1997) procedures. In the first step of the IVID procedure, 1 g of ground ingredient sample was transferred into 100-mL conical flasks and 25 mL of sodium phosphate buffer solution (0.1 M, pH 6.0), and 10 mL of HCl (0.2 M, pH 0.7) were added. To simulate digestion conditions in the stomach, 1 M HCl or NaOH was used to adjust the pH to 2.0, and 1 mL of freshly prepared pepsin solution (10 mg/mL; ≥250 units/mg solid, P7000, Pepsin from porcine gastric mucosa, Sigma-Aldrich, St. Louis, MO, USA) was added to the samples. To avoid bacterial contamination, 0.5 mL of chloramphenicol (C0378, Chloramphenicol, Sigma-Aldrich, St. Louis, MO, USA) solution (5 g/L ethanol) was also added. Test flasks were closed with a silicon stopper and incubated in a shaking incubator at 39°C for 6 h.

After incubation, the second step of the procedure simulated the digestion in the small intestine. Firstly, 10 mL of sodium phosphate buffer solution (0.2 M, pH 6.8) and 5 mL of NaOH (0.6 M, pH 13.8) were added to the samples. Then, pH was adjusted to 6.8 using 1 M HCl or NaOH. Thereafter, 1 mL of freshly prepared pancreatin solution (50 mg/mL; 4 × USP, P1750, Pancreatin from porcine pancreas, Sigma-Aldrich, St. Louis, MO, USA) was added. After incubation in a shaking incubator at 39°C for 18 h, 5 mL of 20% sulfosalicylic acid solution was added and samples were left for 30 min at room temperature to precipitate the indigestible protein. The samples were then filtered through pre-dried and -weighed glass filter crucibles (Filter Crucibles CFE Por. 2, Robu, Hattert, Germany) containing 400 mg of Celite using the Fibertec System (Fibertec System 1021 Cold Extractor, Tecator, Hӧganӓs, Sweden). Test flasks were rinsed twice with 1% sulfosalicylic acid solution, and 10 mL of 95% ethanol and 99.5% acetone were added twice to the glass filter crucibles. Glass filter crucibles with undigested residues were dried at 130°C for 6 h.

The procedure for IVTTD consisted of three-steps. The first and second steps were similar to the IVID procedure, except for the weight of the samples, concentration of the enzymes, and incubation time. For IVTTD, 0.5 g of ingredient sample was used, and the concentrations of pepsin and pancreatin solutions were increased to 25 and 100 mg/mL, while the incubation times were reduced to 2 and 4 h, respectively. In the third step of the IVTTD procedure, 10 mL of 0.2 M EDTA solution was added to the samples. The pH was then adjusted to 4.8 by adding acetic acid 30% or 1 M NaOH. Samples were supplemented with 0.5 mL of multi-enzyme (V2010, Viscozyme^®^ L, Sigma-Aldrich, St. Louis, MO, USA) as a substitute for microbial enzymes, and incubated in a shaking incubator for 18 h at 39°C. After incubation, the undigested residues were collected and dried as previously described in the IVID procedure.

### Calculations and statistical analyses

The IVID or IVTTD of DM (%) was calculated using the following equation:

$${\text{IVID}}\;{\text{or}}\;{\text{IVTTD}}\;{\text{of}}\;{\text{DM}}\;\left( \% \right)\, = \,\left[ {\left( {{\text{DM}}_{\text{TI}} \,{-}\,{\text{DM}}_{\text{RS}} } \right)/{\text{DM}}_{\text{TI}} } \right]\, \times \, 100,$$

where DM_TI_ and DM_RS_ (g) are the weight of DM in the test ingredient and the undigested residue collected from IVID or IVTTD procedure, respectively.

Data were analyzed by MIXED procedure of SAS (SAS Inst. Inc., Cary, NC, USA). The model included EC addition as a fixed variable. Differences between least squares means were determined by the PDIFF option with the Tukey’s adjustment. The significance and tendency of treatment effects were declared at *p* < 0.05 and 0.05 ≤ *p* < 0.10, respectively.

## Results and discussion

The IVID of DM in the nine test ingredients, with or without EC addition, is presented in Table 1. The EC addition increased the IVID of DM in copra meal (*p* = 0.047) and tended to increase the values of corn (*p* = 0.056), wheat (*p* = 0.052), barley (*p* = 0.057), palm kernel meal (*p* = 0.096), cottonseed meal (*p* = 0.096), and DDGS (*p* = 0.084). On the other hand the EC did not affect the IVID of DM in soybean meal and rapeseed meal. Boisen and Fernández (1995) reported the in vitro indigestibility of DM in 17 feed ingredients and found that the IVID of DM in wheat and barley were 86.4 and 79.7%, respectively, which are values similar to the ones obtained in the present study. Among protein sources, palm kernel meal, cottonseed meal, and copra meal showed quite low IVID of DM. This seems to be due to a relatively high level of fiber content in these ingredients (Noblet and Perez 1993). It has been shown that copra meal contains high level of NSPs, especially hemicellulose, which is a substrate for carbohydrases such as xylanase (Choct 1997; NRC 2012). This might be the reason for the increase of IVID of DM in copra meal with EC addition.

**Table 1:** In vitro ileal digestibility of dry matter in test ingredients, with or without the addition of an enzyme complex

Item	Ileal digestibility, %		SEM^b^	*p* value
	Control	Enzyme complex addition^a^		
Corn	83.0	84.1	0.30	0.056
Wheat	87.4	88.1	0.17	0.052
Barley	75.5	76.8	0.36	0.057
Soybean meal	73.1	75.7	7.43	0.282
Rapeseed meal	64.5	64.7	0.36	0.729
Palm kernel meal	29.3	31.4	0.68	0.096
Cottonseed meal	46.5	48.8	0.75	0.096
Copra meal	47.0	48.5	0.37	0.047
DDGS^c^	61.6	62.6	0.32	0.084

Each least squares mean represents three observations.

^a^The enzyme complex was added at 1.0% and consisted of xylanase, protease, and phytase (100, 700 and, 300 units/g, respectively).

^b^Standard error of the mean.

^c^Distillers dried grains with solubles.

The IVTTD of DM in the test ingredients was not affected by the addition of EC except for cottonseed meal which showed a tendency (*p* = 0.053) for greater IVTTD of DM by the EC addition (Table 2). The effects of EC addition might have been diluted by the addition of Viscozyme^®^ containing a wide range of multi-carbohydrases, in the third step of the procedure. For example, Boisen and Fernández (1997) reported an increase of organic matter degradation in soybean meal with the addition of Viscozyme^®^ indicating a higher content of fermentable fiber in soybean meal. However, Viscozyme^®^ addition in the present study most probably did not completely reflect the microbial activity in the hindgut. Although we reduced the amount of sample from 1.0 to 0.5 g to avoid possible underestimation of IVTTD (as previously observed for protein-rich feedstuffs; Boisen and Fernández 1997), the difference between IVID and IVTTD of DM in protein sources ranged from 3.4 (copra meal) to 4.3 (DDGS) percentage units. This difference was much smaller than the one reported in the study by Boisen and Fernández (1997; approximately 20% units).

**Table 2:** In vitro total tract digestibility of dry matter in test ingredients, with or without the addition of an enzyme complex

Item	Total tract digestibility, %		SEM^b^	*p* value
	Control	Enzyme complex addition^a^		
Corn	86.2	87.7	0.74	0.231
Wheat	90.7	91.7	0.45	0.185
Barley	78.4	78.7	0.60	0.699
Soybean meal	77.1	78.8	1.15	0.374
Rapeseed meal	68.2	68.7	0.53	0.504
Palm kernel meal	32.8	33.6	1.32	0.693
Cottonseed meal	50.6	52.1	0.38	0.053
Copra meal	50.4	51.3	0.66	0.396
DDGS^c^	65.9	66.9	0.49	0.231

Each least squares mean represents three observations.

^a^The enzyme complex was added at 1.0% and consisted of xylanase, protease, and phytase (100, 700, and 300 units/g, respectively).

^b^Standard error of the mean.

^c^Distillers dried grains with solubles.

## Conclusions

In conclusion, the effects of EC addition on the IVID or IVTTD of DM may vary, depending on the test ingredient and method used.
